# Supplementary material for: Genetic Progression and the Waiting Time to Cancer
Source: PLoS Comput Biol. 2007 Nov 9;3(11):e225. doi: 10.1371/journal.pcbi.0030225 (PMC2065895; doi:10.1371/journal.pcbi.0030225)
Supplement: Supplementary file 1 [file pcbi.0030225.sd001.pdf]

# Analytical approximations for the expected waiting time

(Supporting information for N. Beerenwinkel, T. Antal, D. Dingli, A. Traulsen, K. W. Kinzler, V. E. Velculescu, B. Vogelstein, M. A. Nowak, **Genetic progression and the waiting time to cancer**)

We derive analytical approximations for the expected waiting time for a cell with $k$ mutations to appear. We consider the Wright-Fisher process for constant population size: we define the model in Section I, in Section II we present a simple argument which works only for weak selection, then in Section III we develop an approximation for strong selection. Finally in Section IV growing cell populations are investigated.

## I. WRIGHT-FISHER PROCESS

Consider a population with a constant number $N$ of cells. In every cell division mutations occur at rate $u$ per locus. Each cell has $d$ susceptible loci, and a mutation at each locus increases fitness by the same amount $s$. Thus, when $j$ of the loci are mutated, the fitness of the cell is proportional to $(1+s)^j$. Let $N_j = N_j(t)$ be the number of cells with $j$ mutations out of the $d$ susceptible loci at time $t$, and $x_j = N_j/N$ be their relative frequency. We assume that the system evolves according to the Wright-Fisher model [1], where cells evolve in non-overlapping generations, and each cell independently chooses a parent cell from the previous generation with a probability proportional to the fitness of the parent. Each cell becomes identical to its parent apart from mutations which occur with probability $u$ at each unmutated gene location. Consequently the probability of a configuration $[N_0(t+1), \dots, N_d(t+1)]$ is given by the multinomial distribution

$$\frac{N!}{N_0(t)! \cdots N_d(t)!} \prod_{j=0}^{d} \theta_j^{N_j(t)} \tag{1}$$

with parameters

$$\theta_j = \sum_{i=0}^{j} \binom{d-i}{j-i} u^{j-i} (1-u)^{d-j} \frac{(1+s)^i x_i}{\sum_\ell (1+s)^\ell x_\ell}. \tag{2}$$

The parameter $\theta_j$ is the probability that a cell in the next generation will have $j$ mutations. If the mutation rate is small $u \ll 1$ we can neglect multiple mutations, and $\theta_j$ simplifies to

$$\theta_j = \frac{(1+s)^j x_j}{\sum_\ell (1+s)^\ell x_\ell} + u(d-j+1) \frac{(1+s)^{j-1} x_{j-1}}{\sum_\ell (1+s)^\ell x_\ell}.$$

The first term is the probability to produce an additional cell of type $j$ without mutation, while the second term is the probability that a cell of type $j-1$ mutates and produces a cell of type $j$. In the simulations we did not need to use this approximation.

## II. DETERMINISTIC APPROACH

In the large $N$ limit we may try to neglect stochastic fluctuations in order to obtain a deterministic equation [1]. We also assume that $u$ and $s$ are small, hence we only keep their leading order behavior. Considering $x_j(t)$ as a continuous variable in time we arrive at a system of ordinary differential equations

$$\dot{x}_j = u\left[(d-j+1)x_{j-1} - (d-j)x_j\right] + s x_j (j - \langle j \rangle) \tag{3}$$

where the dot represents the time derivative, and $\langle j \rangle$ is the average number of mutant loci at a given time,

$$\langle j \rangle = \sum_i i\, x_i(t). \tag{4}$$

The terms on the right hand side of (3) are easy to interpret. The first (gain) term describes cells with $j-1$ mutations becoming cells with $j$ mutations by acquiring a new mutation at one of the $(d-j+1)$ possible loci. The second (loss) term similarly accounts for cells with $j$ mutations undergoing a new mutation at one of the $(d-j)$ possible loci. The last term describes the effect of fitness, where each sub-population grows with a rate of their fitness advantage compared to the average fitness. Note also that densities remain normalized $\sum_j x_j = 1$ due to the $\langle j \rangle$ term.

We are interested in the time until the first cell with $k$ mutations appears, i.e., until $x_k = 1/N$. If $k \ll d$, the number of available mutations is approximately $d$, and we have

$$\dot{x}_j = ud(x_{j-1} - x_j) + s x_j (j - \langle j \rangle), \tag{5}$$

a somewhat simpler system of coupled first order differential equations. The full solution is the Poisson distribution with the time dependent parameter $\lambda = \lambda(t)$,

$$x_j = \frac{\lambda^j e^{-\lambda}}{j!}, \quad \lambda = \frac{ud}{s}(e^{st} - 1). \tag{6}$$

This solution can be easily verified by substituting it back into (5). This solution describes a distribution with equal mean position and variance

$$\langle j \rangle = \operatorname{var} j = \lambda, \tag{7}$$

both growing exponentially in time for generic parameter values.

This behavior, however, is not supported by simulations, where we observe a traveling wave solution with constant speed and constant width (see Fig.1). The reason for the failure of this replicator description is the

following. The deterministic equation produces all types of mutants instantaneously, which then start to multiply, especially the ones with many mutations. This makes the distribution over $j$ (or over $t$) much wider then in the simulations. In other words, $N_0$ is large enough for the deterministic equation to predict $N_1$ correctly, but then $N_1$ is relatively small when the first cell with $j = 2$ mutations arrives. Hence the fluctuations cannot be neglected, and the deterministic description fails to predict $N_2$ correctly.

Note, however, that without selection, *i.e.* for $s \to 0$ and $\lambda \to udt$, equation (6) becomes a good approximation. In this case, the time $t_k$ to reach a $k$-fold mutant can be expressed from the condition $x_k(t_k) = 1/N$, as

$$t_k = \frac{-k}{ud}\, W\left[-\frac{k!^{1/k}}{kN^{1/k}}\right] \qquad \text{for } s \to 0, \tag{8}$$

where the Lambert $W$ function is the inverse function of $f(x) = xe^x$ [2]. For example for $N = 10^9$ and $ud = 10^{-5}$ it gives $t_{20} \approx 3.5 \times 10^5$, while simulations result in $t_{20} \approx 5.6 \times 10^5$. For positive selection $s > 0$, however, we need to develop an alternative approximation, which we do in the next section.

## III. WAVE-LIKE SOLUTION

Inspired by simulation results, we now develop a better approximation for the waiting time $t_k$. We decouple the evolution due to selection from the evolution due to mutation. We model the selection part as a deterministic process, but treat mutations stochastically.

First we consider only selection. For cell types already present in the system, we neglect the effect of mutation in the time evolution, since usually $s \gg ud$. Then the governing equation (3) simplifies to

$$\dot{x}_j = s x_j (j - \langle j \rangle), \tag{9}$$

where we extend the range of $j$ to all integers. This equation has a Gaussian traveling wave solution

$$x_j = A \,\exp\left[-\frac{(j - vt)^2}{2\sigma^2}\right], \tag{10}$$

with constant speed $v$, and constant width $\sigma$. A continuously varying $j$ would imply a normalization constant $A = 1/\sqrt{2\pi\sigma^2}$, and we use this value here as an approximation. Substituting solution (10) back into (9) yields a simple relationship between the speed and the width of the traveling wave of mutants,

$$v = s\sigma^2. \tag{11}$$

Now we have to consider the mutations which we have neglected so far. Notice that if we introduce each new type of mutant one after the other at a given speed, we also obtain (after some transient time) the solution (10)

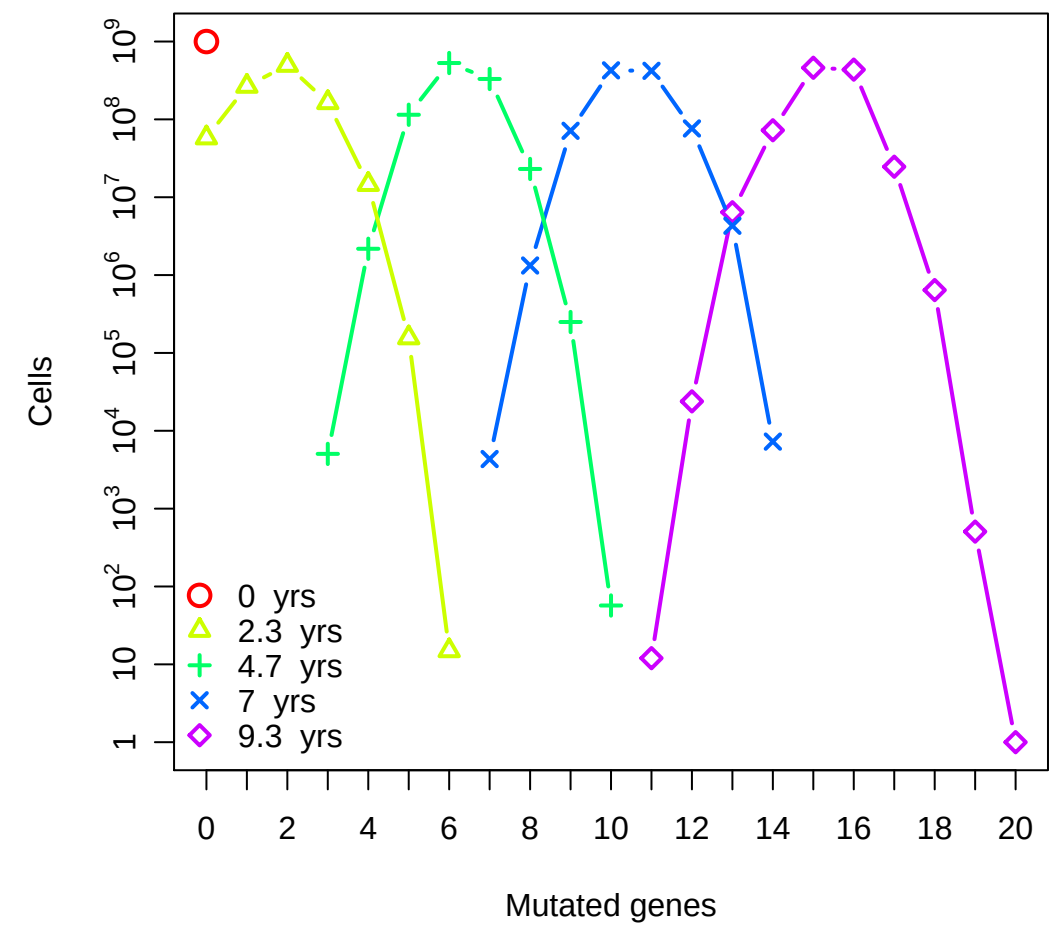


FIG. 1: Simulation results for the distribution of cells with given number of mutated genes at fixed times. The data can be very well approximated by a Gaussian wave traveling at constant speed to the right. The parameters of this simulation were $N = 10^9$, $s = 0.01$, $\mu = 10^{-7}$, $d = 100$, and generation time of one day.

with the width given by (11). Simulations of the Wright-Fisher process support that $x_j(t)$ is a Gaussian (after an initial transient phase), that it has a constant width (see Fig. 1), and that the relationship (11) between the width and the speed holds.

Let us now derive an approximate expression for the speed $v$ of the mutant wave in the stationary state. We need to know the average time $\tau$ at which the first new cell with $j + 1$ mutations appears after the birth of the first cell with $j$ mutations. We assume that $\langle j \rangle$ does not change during this short time, and define the constant $\gamma = j - \langle j \rangle$. From (9) the density $x_j$ initially grows exponentially in time [3],

$$x_j(t) = \frac{1}{N} e^{s\gamma t}, \tag{12}$$

where we also set $x_j(0) = 1/N$, as we start from a single mutant. We approximate the time $\tau$ as the time until, on average, one mutant is produced [4],

$$Nud \int_0^{\tau} x_j(t)dt = ud \int_0^{\tau} e^{s\gamma t} dt = \frac{ud}{s\gamma}(e^{s\gamma\tau} - 1) = 1 \;,$$

which leads to the speed of the mutant wave

$$v = \frac{1}{\tau} = \frac{s\gamma}{\log\left(1 + \dfrac{s\gamma}{ud}\right)} \approx \frac{s\gamma}{\log \dfrac{s\gamma}{ud}} \;. \tag{13}$$

As $\gamma$ is typically of order one in our simulations, we assumed here that $s\gamma \gg ud$ is also true in the $s \gg ud$ limit.

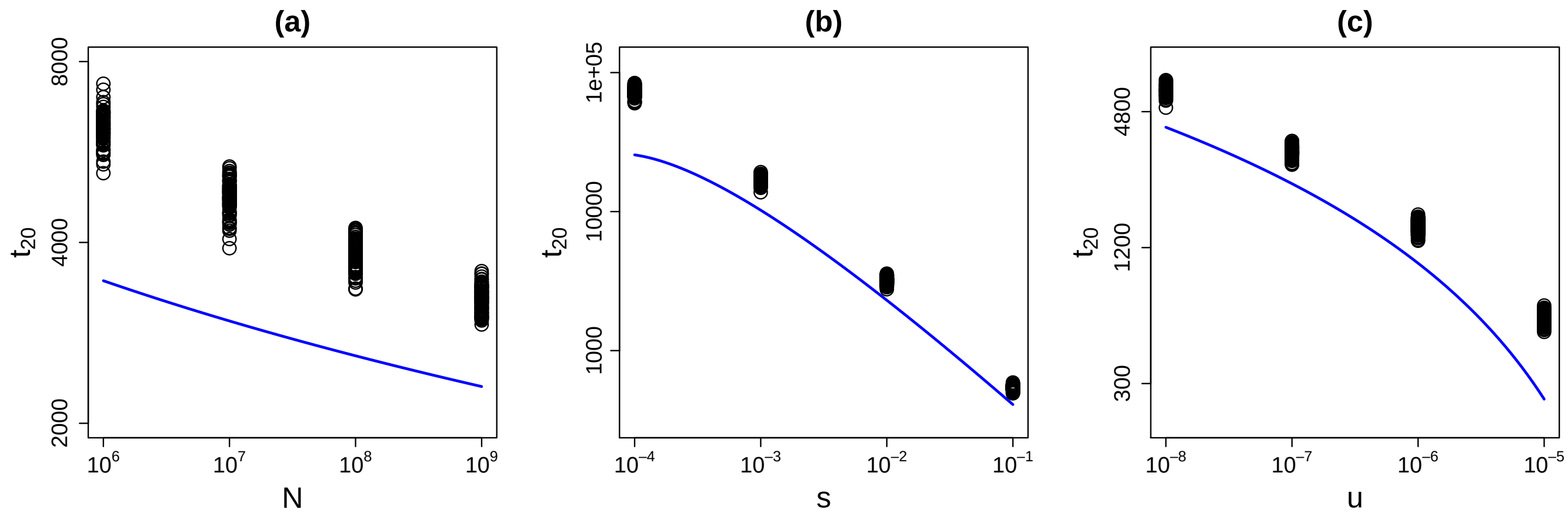


FIG. 2: Expected waiting time for a cell with 20 mutantions, $t_{20}$, as a function of (a) the population size $N$, (b) the selective advantage $s$ per mutation, and (c) the per-locus mutation rate $u$. The circles are the results of 100 independent simulations at each parameter set. We always assumed $d = 100$ sensitive loci, and set $N = 10^9$ in (b) and (c), $s = 0.01$ in (a) and (c), and $u = 10^{-7}$ in (a) and (b). The solid curves correspond to the analytic approximation (17).

Next, we determine $\gamma$. Since $vt = \langle j \rangle$, at the moment when there is exactly one $j$ cell, we have from (10) that

$$\frac{1}{\sqrt{2\pi\sigma^2}} \exp\left(-\frac{\gamma^2}{2\sigma^2}\right) = \frac{1}{N} \tag{14}$$

and hence

$$\gamma = \sqrt{2}\sigma\sqrt{\log\frac{N}{\sqrt{2\pi\sigma^2}}} \approx \sqrt{2}\sigma\sqrt{\log N} = \sqrt{\frac{2v}{s}\log N}\ .$$

As $\sigma$ is of order one, here we neglected $\log\sqrt{2\pi\sigma^2}$ next to $\log N$, and we also used (11) in the last step. Substituting $\gamma$ into expression (13) for the speed we obtain

$$v = \frac{2s\log N}{\left[\log\left(\frac{s}{ud}\sqrt{\frac{2v}{s}\log N}\right)\right]^2}\ . \tag{15}$$

In the denominator we still have $v$ inside the logarithm, which we approximate by the leading behavior $v \approx s$ to arrive at

$$v \approx \frac{2s\log N}{\left(\log\frac{s}{ud} + \frac{1}{2}\log\log N^2\right)^2} \approx \frac{2s\log N}{\left(\log\frac{s}{ud}\right)^2}\ , \tag{16}$$

where we also neglected the double logarithm term in the last step. This is our final formula for the speed of the wave. Using this expression for the speed we approximate the expected waiting time for the first $k$-fold mutant cell to appear as

$$t_k \approx \frac{k}{v} \approx k\,\frac{\left(\log\frac{s}{ud}\right)^2}{2s\log N} \tag{17}$$

In Figure 2, the dependence of $t_k$, for $k = 20$, on $N$, $s$, and $u$ is analyzed by simulations of the Wright-Fisher model. The simple analytic argument given here leads to the appealing expression (17) for the expected waiting time, which is in good qualitative agreement with the simulation results for the Wright-Fisher process.

## IV. GROWING POPULATION

Let us now study a population which grows exponentially from an initial size $N_{\rm init}$ to a final size $N_{\rm fin}$ during the evolution, that is $N(t) = N_{\rm init}e^{bt}$, where $b$ is chosen such that $N(t_k) = N_{\rm fin}$. For the relative frequencies $x_j$, equation (10) is still valid, but the speed of the wave is no longer constant. Since the speed depends logarithmically on system size [see (16)], it grows linearly in time

$$v(t) = a\log N(t) = a(bt + \log N_{\rm init}) \tag{18}$$

where $a = 2s/[\log(s/ud)]^2$ is a constant. Hence the time at which the wave front reaches $k$ mutations is given by

$$k = \int_0^{t_k} v(\tau)d\tau = at_k\frac{\log N_{\rm init} + \log N_{\rm fin}}{2} \tag{19}$$

which leads to

$$t_k \approx k\,\frac{\left(\log\frac{s}{ud}\right)^2}{s\log N_{\rm init}N_{\rm fin}} \tag{20}$$

for the waiting time for the $k$-fold mutant to appear. Note that this is also the waiting time in a constant population (17) with an effective population size $N_{\rm e} = \sqrt{N_{\rm init}N_{\rm fin}}$. Effective population sizes are frequently used in exponentially growing populations evolving according to the Wright-Fisher model [5].

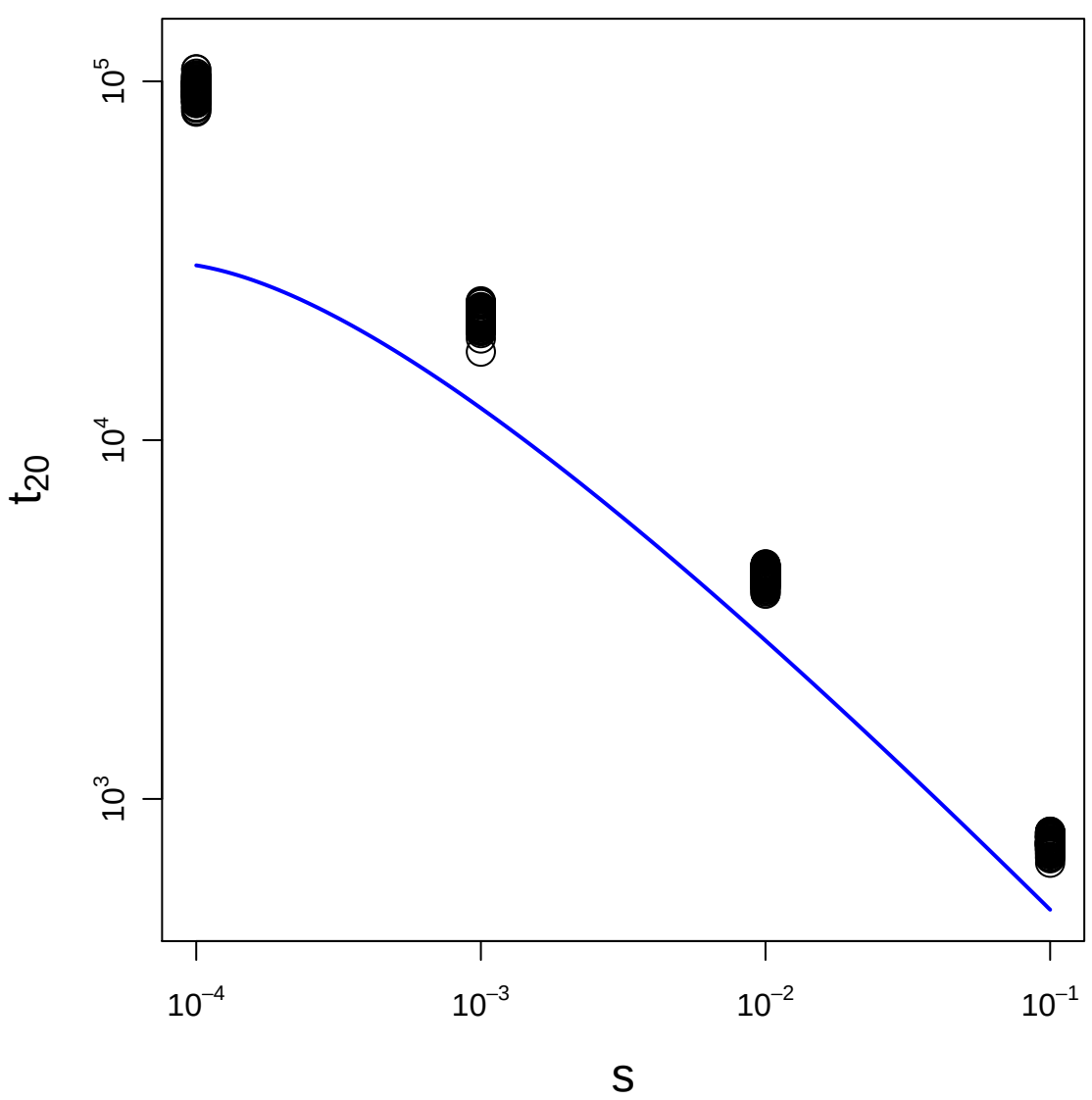


FIG. 3: Expected waiting time for a cell with $k = 20$ mutations, $t_{20}$, as a function of selection strength $s$, in a population which grows exponentially from size $10^6$ to $10^9$. The circles are simulation results of 100 runs for each $s$ value, with mutation rate $u = 10^{-7}$ and $d = 100$. The solid curve is our analytical approximation (20).

In Figure 3 we compare the above formula to simulation results for a growing population. We conclude that our approximation works remarkably well also for growing populations.

[1] Ewens, W. J. (2004) *Mathematical Population Genetics.* Springer, New York.

[2] Weisstein, Eric W. "Lambert W-Function." From MathWorld–A Wolfram Web Resource. http://mathworld.wolfram.com/LambertW_Function.html

[3] Note that $x_j$ eventually deviates from the early exponential growth and follows the Gaussian given by (10).

[4] More precisely we should take into account that a mutant survives only with a finite probability $\rho$, hence we should wait for $1/\rho$ mutants to appear in average. On the other hand the form assumed in (12) for the exponential growth is valid if we average over all possible trajectories, including mutants that go extinct. To obtain the average number of mutants under the condition that they survive we should multiply this expression also by $1/\rho$. Eventually, we have to multiply both sides of the condition (III) by $1/\rho$, which leaves the equation unchanged.

[5] Durrett, R. (2002) *Probability Models of DNA sequence evolution*, Springer-Verlag, New York.
