# Supplementary material for: Genetic Progression and the Waiting Time to Cancer
Source: PLoS Comput Biol. 2007 Nov 9;3(11):e225. doi: 10.1371/journal.pcbi.0030225 (PMC2065895; doi:10.1371/journal.pcbi.0030225)
Supplement: Supplementary file 2 [file pcbi.0030225.sg001.pdf]

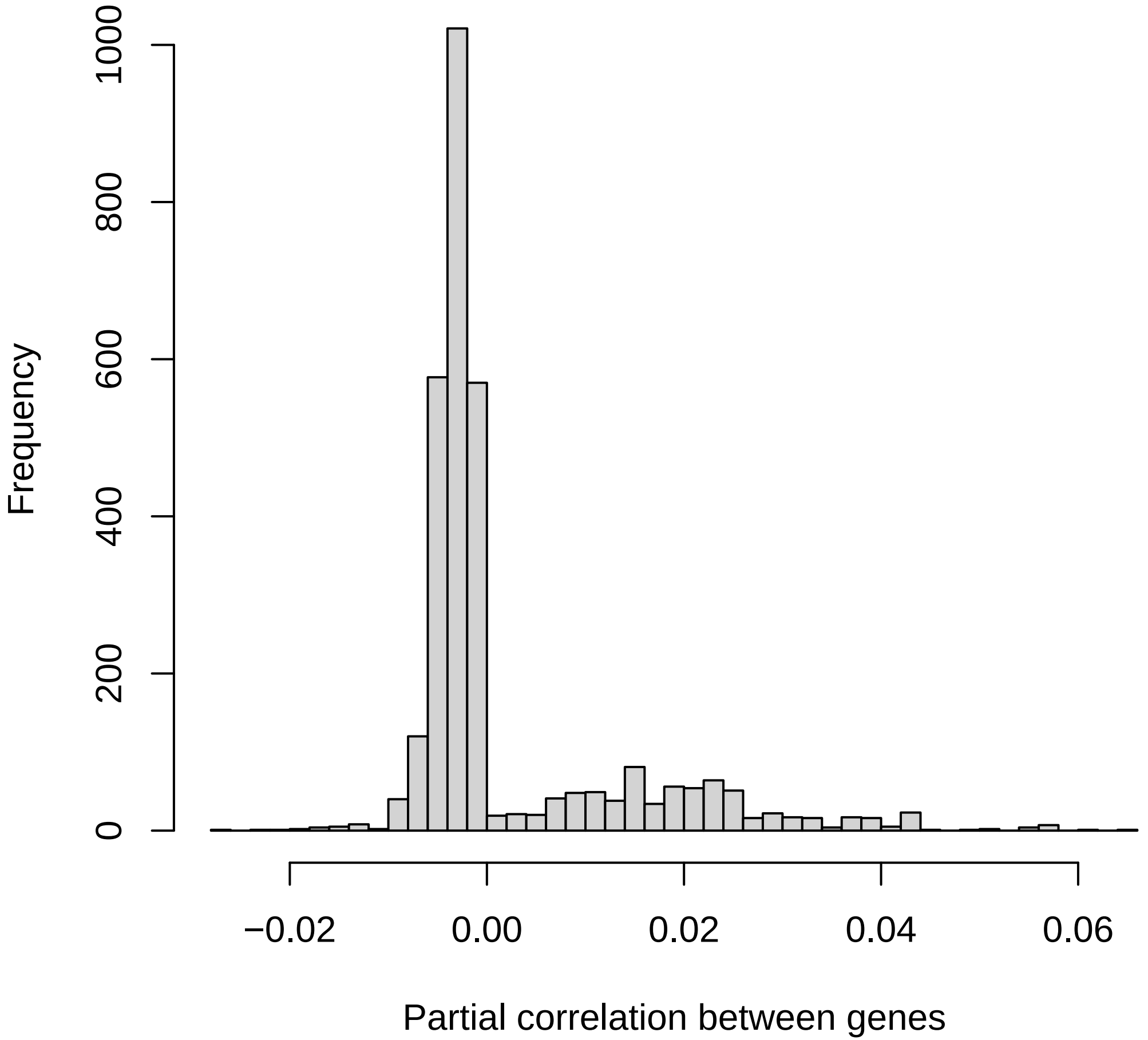

Frequency
0
200
400
600
800
1000
−0.02
0.00
0.02
0.04
0.06
Partial correlation between genes
