# Supplementary material for: Genetic Progression and the Waiting Time to Cancer
Source: PLoS Comput Biol. 2007 Nov 9;3(11):e225. doi: 10.1371/journal.pcbi.0030225 (PMC2065895; doi:10.1371/journal.pcbi.0030225)
Supplement: Supplementary file 3 [file pcbi.0030225.sg002.pdf]

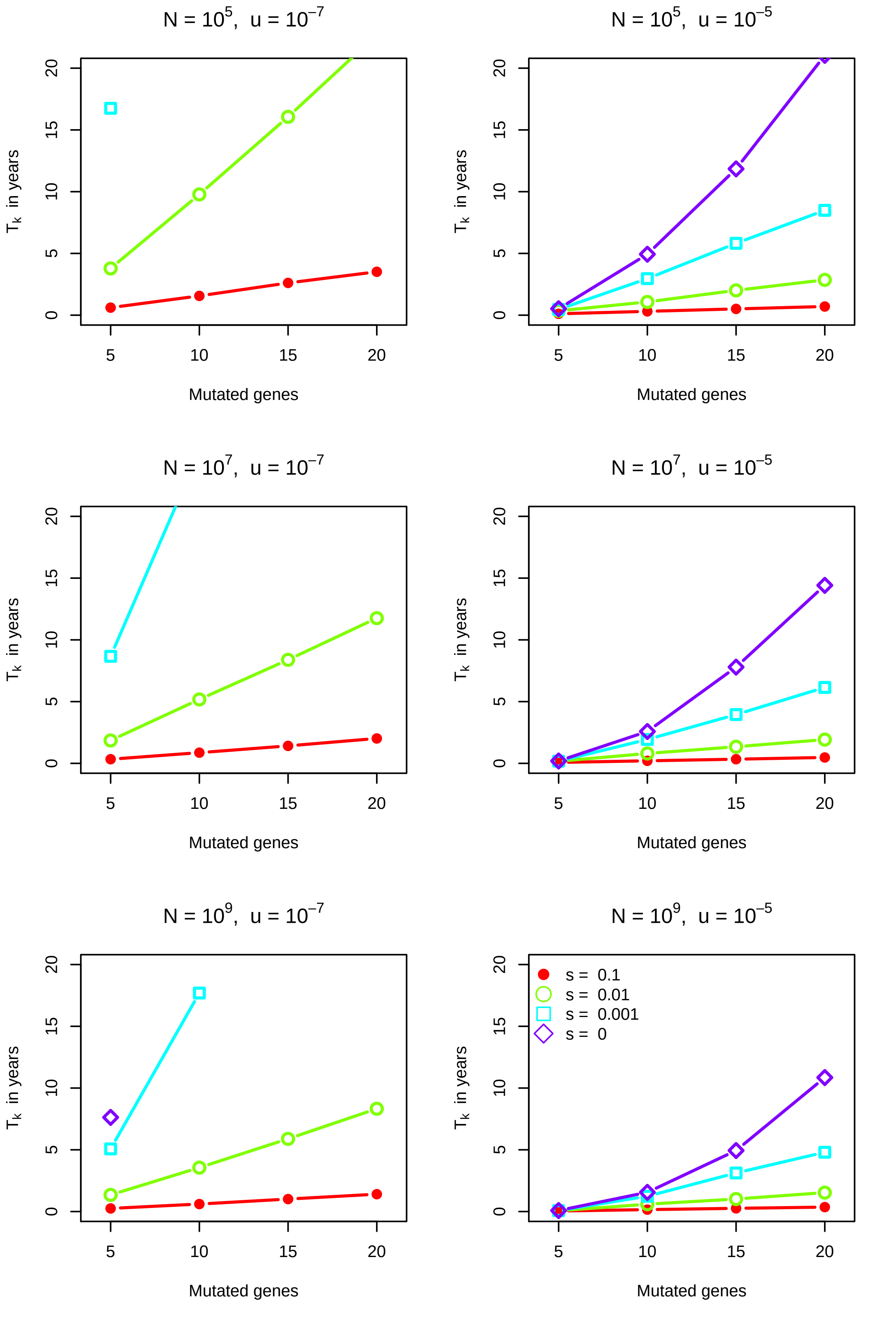

N = 10^5, u = 10^−7
N = 10^5, u = 10^−5
N = 10^7, u = 10^−7
N = 10^7, u = 10^−5
N = 10^9, u = 10^−7
N = 10^9, u = 10^−5
T_k in years
Mutated genes
0
5
10
15
20
s = 0.1
s = 0.01
s = 0.001
s = 0
